# Human MAGI1 expression in endothelial cells protects from the development of localized and systemic scleroderma in mice

**DOI:** 10.1186/s13075-026-03777-y

**Published:** 2026-03-11

**Authors:** Oriana Coquoz, Léa Schlunke, Ilayda Turan, Coralie Hoffmann Schreiner, Isa Uccelli, Cindy Serdjebi, Jimmy Stalin

**Affiliations:** 1 Department of Oncology, Microbiology, and Immunology, University of Fribourg, Chemin du Musée 18, Fribourg, 1700 Switzerland; 2 Biocellvia, 10 Rue Grignan, Marseille, 13001 France

**Keywords:** Localized scleroderma, Systemic sclerosis, Blood vessel, Fibrosis, Angiogenesis, VEGF-A, MAGI1

## Abstract

**Background:**

Systemic sclerosis and localized scleroderma are rheumatoid fibrotic diseases characterized by altered blood vessel permeability and dysregulated angiogenesis. MAGI1 is an intracellular protein expressed at the epithelial and endothelial cell junction, regulating cell adhesion and epithelial/endothelial integrity. Endothelial cell MAGI1 regulates angiogenic function and blood flow. In the present study, we investigated the role of transgenic human MAGI1 in endothelial cells in animal models of localized scleroderma and systemic sclerosis.

**Methods:**

Wild-type and human MAGI1-overexpressing endothelial cells in mice were challenged with sodium hypochlorite or bleomycin injections to induce sclerosis/scleroderma, mimicking the human disease. Conventional methods and automated software were employed to investigate the integrity of lung and skin tissue and quantify fibrosis. In vivo permeability and in vitro angiogenic models were employed to investigate the regulation of endothelial cell function.

**Results:**

Dermal thickening, lung tissue density, fibrotic foci development, and collagen accumulation induced by Bleomycin or sodium hypochlorite were significantly reduced in mice overexpressing human MAGI1 in endothelial cells relative to wild-type mice. Skin permeability and in vitro endothelial cell permeability were also reduced under MAGI1 overexpression. Furthermore, MAGI1 overexpression reduced the in vitro proangiogenic effects of VEGF-A, providing a putative mechanism of action.

**Conclusions:**

The results indicate that endothelial cells expressing transgenic human MAGI1 attenuate tissue permeability and fibrotic responses in models of bleomycin- and sodium hypochlorite-induced scleroderma/sclerosis. MAGI1 overexpression blocks VEGF-A-induced permeability and angiogenesis in endothelial cells. Therefore, restoring endothelial cells could potentially alleviate VEGF-A-driven diseases such as localized scleroderma and systemic sclerosis.

**Supplementary Information:**

The online version contains supplementary material available at 10.1186/s13075-026-03777-y.

## Background

The vascular endothelium is the inner lining of blood vessels. It plays a crucial role in biomechanical and humoral signal transduction to the vessel wall and the surrounding tissue [1, 2]. The inability of the endothelium to modulate these responses and maintain a normal homeostatic state is a hallmark in the development of tissue inflammation and inflammatory pathologies, including localized (i.e., localized scleroderma (LSc) and skin scarring) or systemic inflammatory disorders (i.e., systemic sclerosis (SSc) and lung fibrosis) [2–4]. LSc and SSc are autoimmune diseases characterized by fibrosis of the skin and/or multiple internal organs (e.g., lungs, kidneys) [5]. The pathophysiological process involves the induction of an oxidative burst leading to oxidative stress [6]. Notably, ROS accumulation and subsequent endothelial cell activation [7], as well as chronic inflammation, autoimmunity, and fibroblast activation, contribute to extracellular collagen deposition, leading to tissue scarring and fibrosis [8, 9]. LSc and SSc are associated with tissue fibrosis, for example, in the kidneys and lungs. The etiologies of LSc and SSc are not yet fully established. From the current clinical and experimental evidence, the severity, evolution, and disease outcome are highly heterogeneous [10, 11]. In addition, the late onset of symptoms — often years after exposure to stimuli — makes these diseases difficult to detect and treat early. LSc and SSc begin as localized or systemic inflammatory events that lead to autoimmune disorders and microcirculatory vascular injuries [12, 13]. Scleroderma is described as an autoimmune collagen vascular disease; the most striking feature may be systemic vasculopathy. The subsequent activation of EC leads to the production of large amounts of endothelin-1, which causes vasoconstriction and fibroblast activation, accelerating vascular remodeling and ultimately obliterating small vessels. Indeed, data from animal models have shown that chronic oxidative stress in the skin, induced by bleomycin, a pro-oxidative agent, suffices to induce fibrosis, vascular perturbation, and autoimmunity [6]. Therefore, a better understanding of SSc pathogenesis is needed to identify new molecular targets and develop alternative therapeutic approaches [5].

Recent investigations have highlighted the physiological role of the MAGUK family member with an inverted domain structure 1 (MAGI1) in endothelial cell functions [14]. MAGI1 consists of six PSD95/DiscLarge/ZO-1 (PDZ) domains, a guanylate kinase domain, and two WW domains flanked by two PDZ domains, and participates in the recruitment of cytoplasmic molecules at cell-cell contacts in endothelial and epithelial cells [15, 16]. Through its PDZ domains, MAGI1 associates with multiple PDZ-binding proteins at adherent junctions, including δ-catenin and β-catenin, in epithelial cells. In endothelial cells, MAGI1 has been reported to mediate Rap1 activation through VE-cadherin-dependent cell-cell contact [14]. In vitro laminar flow increases MAGI1, KLF4, and active eNOS (P-eNOS Ser1177) expression in human umbilical vein endothelial cells (HUVECs), and MAGI1 overexpression increases KLF4, Ser1177-P-eNOS levels, and NO production in response to fluid shear stress. In contrast, MAGI1 knockdown (shMAGI1) significantly decreased the level of P-eNOS and abolished eNOS phosphorylation and KLF4 induction [17]. At a cellular level, MAGI1 localizes into large, mature focal adhesions in HUVECs and regulates focal adhesion dynamics. MAGI1 silencing in HUVECs reduces focal adhesion formation and maturation, cell spreading, and actin stress fiber formation. Consistent with these findings, MAGI1 promotes the integrin-dependent adhesion of HUVECs to the ECM and reduces in vitro HUVEC invasion and angiogenic ability [18].

The role of blood vascular endothelial MAGI1 in vascular physiology has only been addressed in recent years. However, little is known about the involvement of endothelial cell MAGI1 in vascular, inflammatory, and fibrotic pathologies. Given the role of MAGI1 in physiopathology and the significant participation of blood vascular endothelial cells in disease development, we investigated the effect of endothelial cell MAGI1 overexpression on the development of tissue inflammation and fibrosis.

## Materials and methods

### Animals

For all the in vivo experiments, FVB/N VEC_tA2::tet_MAGI1 mice (DT) and wild-type single transgenic mice (VEC_tA2 and tet_MAGI1 mice) were used. Animals were treated in accordance with the guidelines of the Swiss and Fribourg cantonal regulations on the care and use of laboratory animals. The protocols were revised and approved by the Animal Care Committee of Canton de Fribourg and the Welfare Animal Officer of the University of Fribourg (authorization numbers 2020_18_FR and 2023-27-FR). Animals were housed and bred in a conventional animal facility at the University of Fribourg. Regarding the animal model, because of resistance to fibrosis development observed in young animals and in female mice, only male mice aged 9–10 weeks have been used.

Blinding was ensured as follows: 1/ mice were randomly given a number before genotyping, 2/ the technician performing the genotyping (PCR and agarose gel migration) was not involved in animal experiments, 3/ the study coordinator gave to the person in charge of the treatments the identification number and corresponding treatment, 4/ the person in charge of the analysis (inflammation, fibrosis, cell counting) is a different person and not in charge of the treatments or a software (MorphoQuant), 5/ at the end the study coordinator linked the identification number of the animal with the results of the tissue analysis.

### Hypochlorite and bleomycin-localized scleroderma models

For the hypochlorite (HOCl) and Bleomycin-LSc mouse models, mice were anesthetized with isoflurane (4% induction, 1.5–2.5% maintenance). HOCl was generated by adding NaClO (9.6% active chlorine) to KH_2_PO_4_ solution (100 mM, pH: 6.2) using a ratio of 1/100. Mice were injected intradermally with HOCl or bleomycin (0.02U/150 µL per site) at two lower back locations daily for 5 weeks. Mice’s body weight and well-being were monitored according to the approved monitoring score sheet. After 5 weeks, injections were discontinued.

### Bleomycin-systemic sclerosis model

Mice were anesthetized with isoflurane (4% induction, 1.5–2.5% maintenance) and then injected intraperitoneally with bleomycin (35 mg/kg/100 µL) five times, every 3 days, over 15 days (days 1, 4, 7, 10, and 13). They were euthanized after two injections, five injections, or 18 days after the fifth injection. Mice’s body weight and well-being were monitored.

### Vascular permeability mile’s assay

For the in vivo permeability assay, the dorsal flank of adult mice (age 6–8 weeks) was bilaterally shaved 24 h before the experiment. The next day, a solution of histamine H1 receptor antagonist pyrilamine maleate salt (4 mg/kg body weight in 0.9% saline) was administered intraperitoneally 30 min before VEGF injection to block the effects of local histamine release resulting from the injection-induced mast cell activation. VEGF (100ng in 50µL, R&D Systems) and PBS were injected intradermally into the dorsal flanks, while bleomycin (20 µg/100 µL PBS) and IL-1α (1 µg/100 µL PBS) were injected during three consecutive days. Then, Evans blue solution (100 µL of 1.5% in sterile saline) was injected into the tail vein and circulated for 30 min. Thirty minutes later, mice were sacrificed by cervical dislocation, and the dorsal skin was excised. The weight of the excised skin tissue was recorded, and the samples were dried overnight by placing them in 1.5 mL tubes in a heating block at 55 °C. Evans blue was extracted from the excised tissue by immersion in formamide for 24 h at 55 °C, and the amount of blue dye was quantified by spectrometry at 620 nm. The weight of the excised tissue was used to normalize the measured optical density of each sample.

### Paraffin embedding and tissue colorations

Tissue samples were fixed in formalin for 24 h at room temperature, washed thoroughly with water, and then stored at 4 °C in ethanol 70%. The tissues were dehydrated through a series of ethanol solutions (100%, 95%, 80%, and then 70%) before being embedded in paraffin wax. Sections of 3–5 μm thickness were cut using a microtome and mounted onto glass slides. Tissue sections were deparaffinized in xylene, rehydrated through alcohol solutions, and stained with hematoxylin and eosin (HE) for general morphology assessment and tissue inflammation score measurement. Picro-Sirius red (PSR) and Masson trichrome stains were used to evaluate collagen deposition and fibrosis. Slides were scanned using a digital slide scanner (Nano Zoomer, Hamamatsu Photonics). H&E and Masson trichrome stains were used for manual analysis of inflammatory and fibrotic areas. In contrast, PSR was used for automated analysis to determine tissue density, assess areas covered by fibrotic foci, and collagen fibers (MorphoQuant, BioCellVia, France).

Cell counting, inflammatory and fibrotic areas, and skin thickness were performed using the NDP.view2 software (Hamamatsu) when tissue sections allowed it. Individual cells (brown coloration) were manually counted within the region of interest using the annotation function. The number of cells in each region was reported per unit area. Inflammatory (based on HE stains) and fibrotic (based on Masson trichrome stains) regions were manually drawn using the annotation function. The inflammatory and fibrotic areas were reported as a percentage of the total tissue area. Finally, skin thickness (based on HE stains) at at least 10 sites on the skin surface was manually drawn using the annotation function.

### Immunostaining

Immunostaining of macrophages (F4/80), Vimentin, myofibroblasts (α-Smooth Muscle Actin), and lymphocytes (CD3) have been performed by the Mouse Pathology Facility at the University of Lausanne/CHUV (Centre hospitalier universitaire Vaudois). References for antibodies used for IHC are anti-CD3 antibody (Abcam, ab5690), anti-F4/80 antibody (Invitrogen, MF48000), anti-Vimentin antibody (Cell Signaling, #5741), and anti-α-Smooth Muscle Actin (Cell Signaling, #19245). Antigen retrieval was performed using EDTA (17 min in a pressure cooker) for the anti-CD3 antibody, proteinase K from DAKO (15 min) for the anti-F4/80 antibody, and citrate solution (microwaved until boiling was initiated, followed by 10 min at a sub-boiling temperature (95°-98 °C) for anti-Vimentin and anti-α-Smooth Muscle Actin antibodies. Incubation with primary antibodies was performed at room temperature for 1 h.

### Hydroxyproline assay

Hydroxyproline is a nonproteinogenic amino acid formed by the enzyme prolyl-4-hydrolase. In animals, hydroxyproline is found almost entirely in collagen; therefore, it is often used as a direct measure of the amount of collagen in the tissue. We used a colorimetric method to determine the amount of hydroxyproline in the tissue. Skin and lung samples collected from mice were processed using the Hydroxyproline Assay Kit (Abcam), in which a brightly colored chromophore is detected by measuring the absorbance at 560 nm with a microplate reader (Tecan Infinite Pro).

### Cell culture

Primary Human umbilical vein endothelial cells (HUVECs) were isolated from human umbilical cords as previously described [18]. HUVEC were cultured in M199 supplemented with Glutamax, 10% FCS, 12 µg/ml of bovine brain extract (Clonetics), 10 ng/ml recombinant epidermal growth factor (EGF) (Genzyme), 25 U/ml heparin, one µg/ml hydrocortisone (Sigma), and 1% penicillin/streptomycin. The ethics committee for human experimentation of Canton Vaud (Switzerland) approved the collection and use of HUVEC (CER-VD_105/04). All cells were maintained in a humidified incubator at 37 °C and 5% CO2.

### Cell transfection

Transient overexpression of MAGI1 in HUVECs (24–48 h) was achieved by using a plasmid encoding human MAGI1 and various transfection systems. The transfection reagents used were Lipofectamine 2000 (Invitrogen), X-tremeGene 9 (Roche), and Fugene 4 K (Promega). Transfected cells were stimulated with human recombinant VEGF-A (20ng/ml) and Wnt3A (100ng/ml) in an M199 medium containing 1% FBS or in complete HUVECs growth medium.

### RNA extraction, cDNA generation, and real time-polymerase chain reaction

RNA was extracted from cells using the NucleoSpin RNA Plus kit (Macherey-Nagel). Cells were scraped after the addition of the LBP lysis buffer to the wells. Cell lysate was loaded onto the gDNA Removal column, and RNA was separated from genomic DNA and other cellular components (e.g., lipids and proteins) until the RNA was eluted. The quality and concentration of the extracted RNA were then analyzed using the NanoDrop 2000/2000c Spectrophotometer. Reverse transcription polymerase chain reaction (RT-PCR) was performed using the high-capacity DNA reverse transcriptase kit to obtain cDNA from the initial RNA.

The generated cDNA was mixed with SYBR Green and primers specific to mouse genes involved in inflammation and fibrosis. The expression of target genes was assessed using quantitative real-time PCR. The values for each gene were normalized to GAPDH by subtracting the CT (cycle threshold) value of GAPDH from the CT value of each gene of interest. The relative expression of each gene in each sample was calculated using the ΔΔCt method. The primers sequences are:

- MAGI1:
  Forward primer: 5’-CAA ACC ACC CTC CAA GCA ATC C-3’
  Reverse primer: 5’-AAG CCA CGA CTG CTT TTC CGC A-3’
- CD31:
  Forward primer: 5’-AAGTGGAGTCCAGCCGCATATC-3’
  Reverse primer: 5’-ATGGAGCAGGACAGGTTCAGTC-3’
- Ve-Cadherin:
  Forward primer: 5’-GAA GCC TCT GAT TGG CAC AGT G-3’
  Reverse primer: 5’-TTT TGT GAC TCG GAA GAA CTG GC-3’
- Alpha-SMA:
  Forward primer: 5’-CTA TGC CTC TGG ACG CAC AAC T-3’
  Reverse primer: 5’-CAG ATC CAG ACG CAT GAT GGC A-3’
- PDGF receptor beta:
  Forward primer: 5’-TGC AGA CAT CGA GTC CTC CAA C-3’
  Reverse primer: 5’-GCT TAG CAC TGG AGA CTC GTT G-3’
- GAPDH:
  Forward primer: 5’-GGA CCT GAC CTG CCG TCT AG-3’
  Reverse primer: 5’-CCA CCA CCC TGT TGC TGT AG-3’

### Protein extraction, gel electrophoresis, and Western blot

Proteins were extracted from cells using the RIPA lysis buffer. The total lysate protein was quantified using BSA as a standard with the Pierce™ BCA Protein Assay Kit (Thermo Fisher Scientific). Absorbance was measured with a microplate reader (Tecan Infinite Pro). Equal amounts of protein were mixed with Laemmli sample buffer (Bio-Rad), and the samples were then separated by SDS-PAGE on 10% polyacrylamide gels (Novex™ WedgeWell™ 10% Tris-Glycine; Invitrogen by Thermo Fisher Scientific) for at least 30 min at 200 V. Proteins were transferred to a nitrocellulose membrane using the Trans-Blot Turbo RTA Midi LF PVDF Transfer Kit (Bio-Rad). Membranes were blocked with 5% milk in Tris-buffered saline tween-20 (TBST) and incubated overnight at 4 °C with primary antibodies. The references of the primary antibodies used for western blotting are: 1/ anti-PDGF receptor β antibody #3162 (Cell signaling antibody), anti-α-smooth muscle actin antibody #19,245 (Cell signaling antibody), anti-β-actin antibody #3700 (Cell signaling antibody), anti-GAPDH antibody #5174 (Cell signaling antibody), and anti-MAGI1 antibody (M5691) (Millipore, Sigma Aldrich). After the washing steps, the HRP-coupled secondary antibodies specific to the primary antibody species were added. They were revealed using a chemiluminescent reaction, and photons were detected using the photon imaging system (iBright™ CL1500 Imaging System).

### In vitro permeability assay

HUVECs were seeded on 0.4 μm pore transwells until reaching 100% confluency. Cells were then stimulated for 24 h. A 70 kDa dextran FITC dye at 10 µg/ml was added to the upper part of the Transwell, and the plates were incubated for 2 h. Then, the lower and upper part of the transwell containing the cell culture medium was taken, and fluorescence (488 and 530 nm) was quantified using a fluorometer.

### Wound healing/migration assay

Wounds were created using a sterile tip in a 12-well plate of HUVECs 24 h after cell transfection, when the cell layer was 100% confluent. Pictures were taken at T = 8 h after the induction medium was added. The wound areas were evaluated using ImageJ software, and the closure area was calculated relative to time zero (T = 0 h).

### MTT viability assay

The MTT (3-[4,5-dimethylthiazol-2-yl]-2,5-diphenyltetrazolium bromide) assay, also known as Thiazolyl Blue, is a colorimetric assay that measures mitochondrial metabolic activity in mammalian cells and serves as an indicator of cell viability. The assay is based on the reduction of the yellow tetrazolium salt 3-(4,5-dimethylthiazol-2-yl)-2,5-diphenyltetrazolium bromide into purple formazan crystals by metabolically active cells. Viable cells contain an NADPH-dependent oxidoreductase that reduces MTT to formazan [19, 20]. The MTT solution was prepared by resuspending 4 mg/mL of powder in PBS. After drug incubation with cells, 50 µL of cell culture medium was removed into another 96-well plate for the LDH assay. Then the remaining cell culture medium was removed, and 50 µL/well of MTT dissolved at 2/5 in PBS was added. The plate was incubated at 37 °C in 5% CO2 for three hours. After incubation, 150 µL of dimethyl sulfoxide (DMSO) was added to each well to dissolve the formazan crystals. The plate was shaken at 200 rpm for 30 min to ensure correct homogenization, and the absorbance was measured at 590 nm and 690 nm using a microplate reader (Tecan Infinite Pro).

### LDH release assay

The lactate dehydrogenase (LDH) assay is a colorimetric method used to determine cell death by measuring the activity of the cytoplasmic enzyme lactate dehydrogenase (LDH) released by damaged cells and accumulating in the cell supernatant. The lactate dehydrogenase enzyme catalyzes the conversion of pyruvate into L-lactate by oxidizing NADH to NAD+, which subsequently reduces iodonitrotetrazolium chloride (INT) to form the colored formazan. This reaction causes a color change from yellow to red, which can be detected by measuring the absorbance at 490 nm. The amount of formazan is directly proportional to the amount of LDH in the supernatant, indirectly indicating the number of damaged or dead cells. The LDH assay was conducted on the supernatant of 96-well plate experiments using the Cytotoxicity Detection Kit PLUS (Roche). At the end of each experiment, 50 µl of the supernatant was carefully transferred to a new 96-well plate. The LDH catalyst was prepared by dissolving the lyophilized catalyst powder in 1mL of ddH2O in 10 min. The LDH catalyst was diluted 1:46 in the dye solution (INT and sodium lactate) immediately before adding it to the supernatant. The plate was incubated at room temperature, protected from the light, for 30 min. After this incubation period, the stop solution was added to each well, and the absorbance was read at 490 nm and 680 nm (background) using a spectrophotometer microplate reader (Tecan Infinite Pro).

### 2D tubulogenic In vitro assay

The angiogenic ability of HUVECs was evaluated using the 2D Matrigel capillary-like structure assay [21]. A film of Matrigel (0.5 mg/ml) was polymerized at 37 °C for 3 hours in the bottom of 96-well plates. HUVECs (10 × 10^3^) were seeded in the wells and incubated for 12 h. Cells were fixed with 4% PFA for 10 min at 4 °C, rinsed with PBS. Pictures were taken using a brightfield microscope at a magnification of 4X, and the number of closed capillary-like structures was counted using the ImageJ software (*n* = 3 replicate wells per condition). Values represent means ± SEM reported as a percentage of control conditions.

### Statistical analysis

The normality of the sample distribution was assessed using the Shapiro–Wilk test. Data from in vitro, ex vivo, and in vivo experiments were analyzed by Student’s t-test or Mann–Whitney test when applicable. Results were considered significant with at least *P* < 0.05 (*), *P* < 0.01 (*), *P* < 0.005 (***), and *P* < 0.001 (****). Results are expressed as mean ± SEM unless otherwise indicated.

## Results

### Human MAGI1 expression in blood vascular endothelial cells protects from localized scleroderma development

**Fig. 1:**
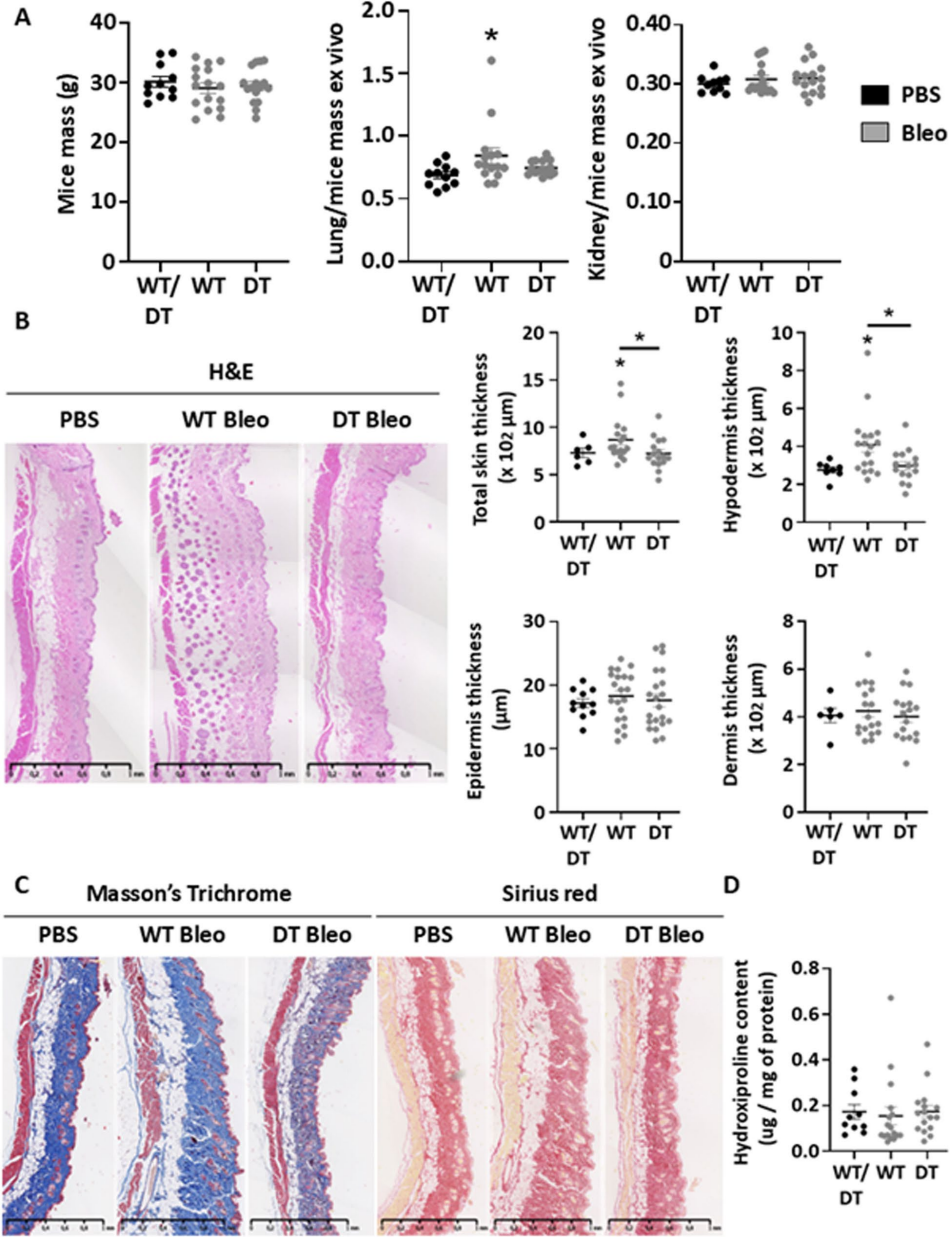
Endothelial cell human MAGI1 overexpressing mice presented attenuated dermal thickening in response to Bleomycin injections. Graph summarizing mice, lung/mice, and kidney/mice mass measurements at euthanasia (**A**). Skin sections of PBS and Bleomycin-treated wild type (WT) and endothelial cell human MAGI1 overexpressing (DT) mice were stained with hematoxylin and eosin. Representative images and data for skin thickness measurements are presented in graphs. Thicknesses were calculated by averaging the measurements from six to ten randomly selected locations in each section using ImageJ (**B**). Representative images of skin sections of PBS and Bleomycin-treated wild-type (WT) and endothelial cell human MAGI1 overexpressing (DT) mice were stained with Masson Trichrome and Sirius Red (**C**). Results from hydroxyproline assay measurements are presented in graphs (**D**). Bars represent the means + SEM. The significance of differences between two groups was evaluated using the unpaired Student’s t-test or Mann–Whitney test. *P < 0.05. (*n* = 11 mice PBS group, 17 mice WT Bleomycin group, 17 mice DT Bleomycin group)

**Fig. 2:**
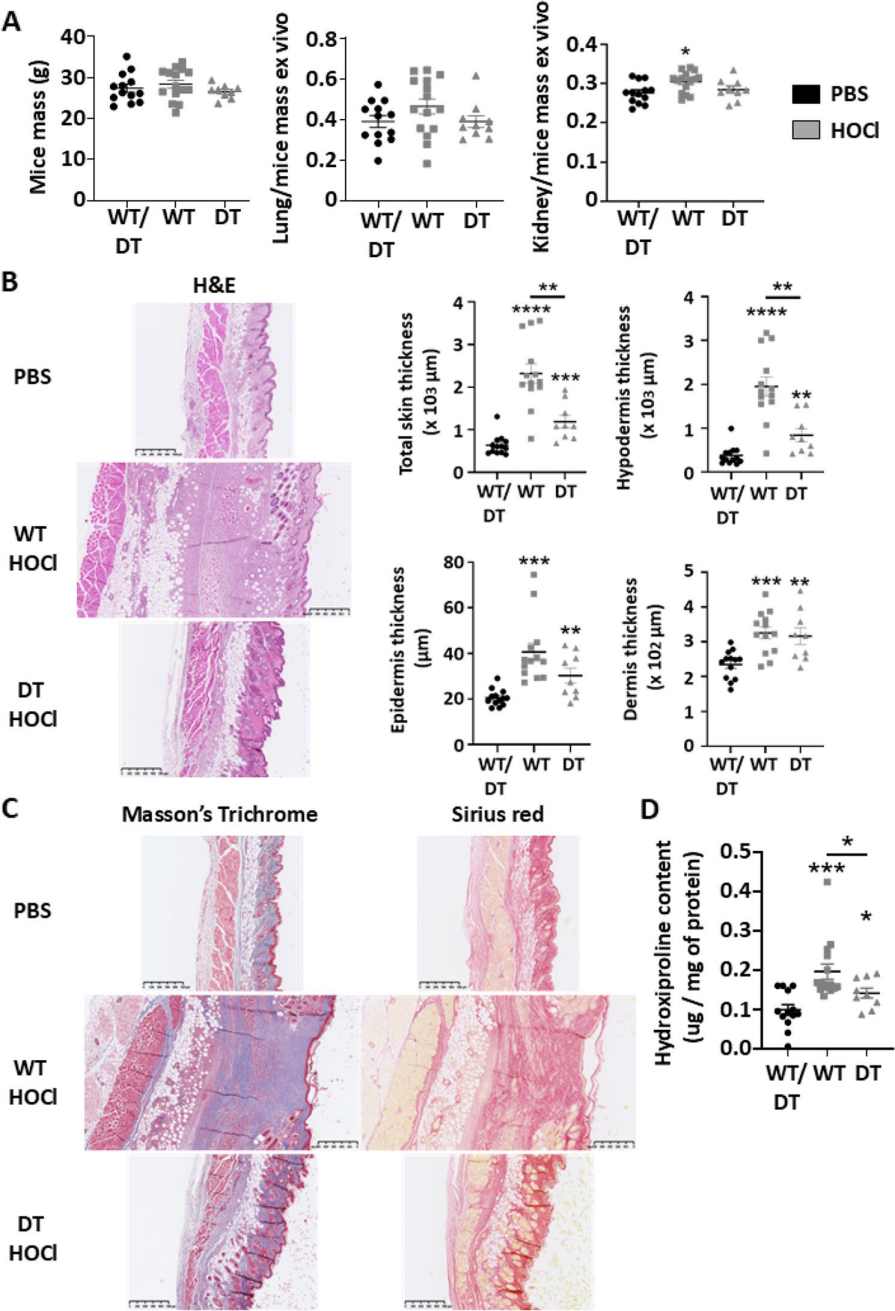
Endothelial cell human MAGI1 overexpressing mice presented attenuated localized scleroderma in response to HOCl injections. Graph summarizing mice, lung/mice, and kidney/mice mass measurements at euthanasia (**A**). Skin sections of PBS and HOCl-treated wild-type (WT) and human MAGI1 overexpressing (DT) endothelial cells from mice were stained with hematoxylin and eosin. Representative images and data for skin thickness measurements are presented in graphs. Thicknesses were calculated by averaging the measurements from six to ten randomly selected locations in each section using ImageJ (**B**). Representative images of skin sections of PBS and Bleomycin-treated wild-type (WT) and endothelial cell human MAGI1 overexpressing (DT) mice were stained with Masson Trichrome and Sirius Red (**C**). Results from hydroxyproline assay measurements are presented in graphs (**D**). Bars represent the means + SEM. The significance of differences between two groups was evaluated using the unpaired Student’s t-test or Mann–Whitney test. *P < 0.05; ** *P* < 0.01; ***P < 0.001. (*n* = 13 mice PBS group/15 mice WT HOCl group/9 mice DT HOCl group)

To investigate the protective role of human MAGI1 in the development of localized scleroderma, we utilized a transgenic mouse model with inducible tissue-specific expression of human MAGI1 (MAGI1-tetOS) in endothelial cells [17, 18]. In this system, endothelial specificity was achieved by expressing the Tet-regulated activator line (VEC_tTA) under a VE-cadherin promoter. LSc was induced in single transgenic mice (WT) and double transgenic mice expressing human MAGI1 on blood vessels (DT) using Bleomycin (Fig. 1) and hypochlorous acid (HOCl) (Fig. 2) relative to PBS injection.

Body weight was affected by neither HOCl nor Bleomycin treatments (Fig. 1A, left, and Fig. 2A, left). However, the ratios of lung/mice mass and kidney/mice mass were significantly higher, respectively, in bleomycin- and HOCl-WT treated mice relative to PBS-treated and bleomycin- and HOCl-DT mice overexpressing human MAGI1 in endothelial cells (Fig. 1A middle and Fig. 2A left).

Hemato-eosin (H&E) coloration was performed on the skin section, and total skin, hypodermis, epidermis, and dermis thickness were measured. We observed that bleomycin increases total skin and hypodermis thickness in WT mice relative to PBS-injected mice. However, it is significantly reduced in DT mice overexpressing MAGI1 in endothelial cells (Fig. 1B). In addition, HOCl treatment increases hypodermis, epidermis, dermis, and total skin thickness in WT mice relative to PBS-injected mice. Interestingly, hypodermis and total skin thickness are significantly decreased in DT mice overexpressing MAGI1 in endothelial cells. In contrast, only a trend to a decrease is observed between ST and DT mice in the epidermis thickness (Fig. 2B). Finally, Trichrome Masson’s (TM) and Sirius Red (SR) colorations, and tissue hydroxyproline quantification assays have been performed to visualize and quantify collagen deposition into the skin. We observed that the collagen fibers and hydroxyproline content remain unchanged in Bleomycin-treated WT mice, whereas they are increased in HOCl-treated WT mice (Figs. 1C and D and 2C and D). Moreover, HOCl-treated DT mice have decreased collagen fiber and hydroxyproline content relative to HOCl-treated WT mice (Fig. 2C and D).

These results demonstrate that human MAGI1 overexpression in endothelial cells protects against skin thickness and collagen deposition in two animal models of localized scleroderma.

### Human MAGI1 expression in blood vascular endothelial cells protects from systemic sclerosis development

**Fig. 3:**
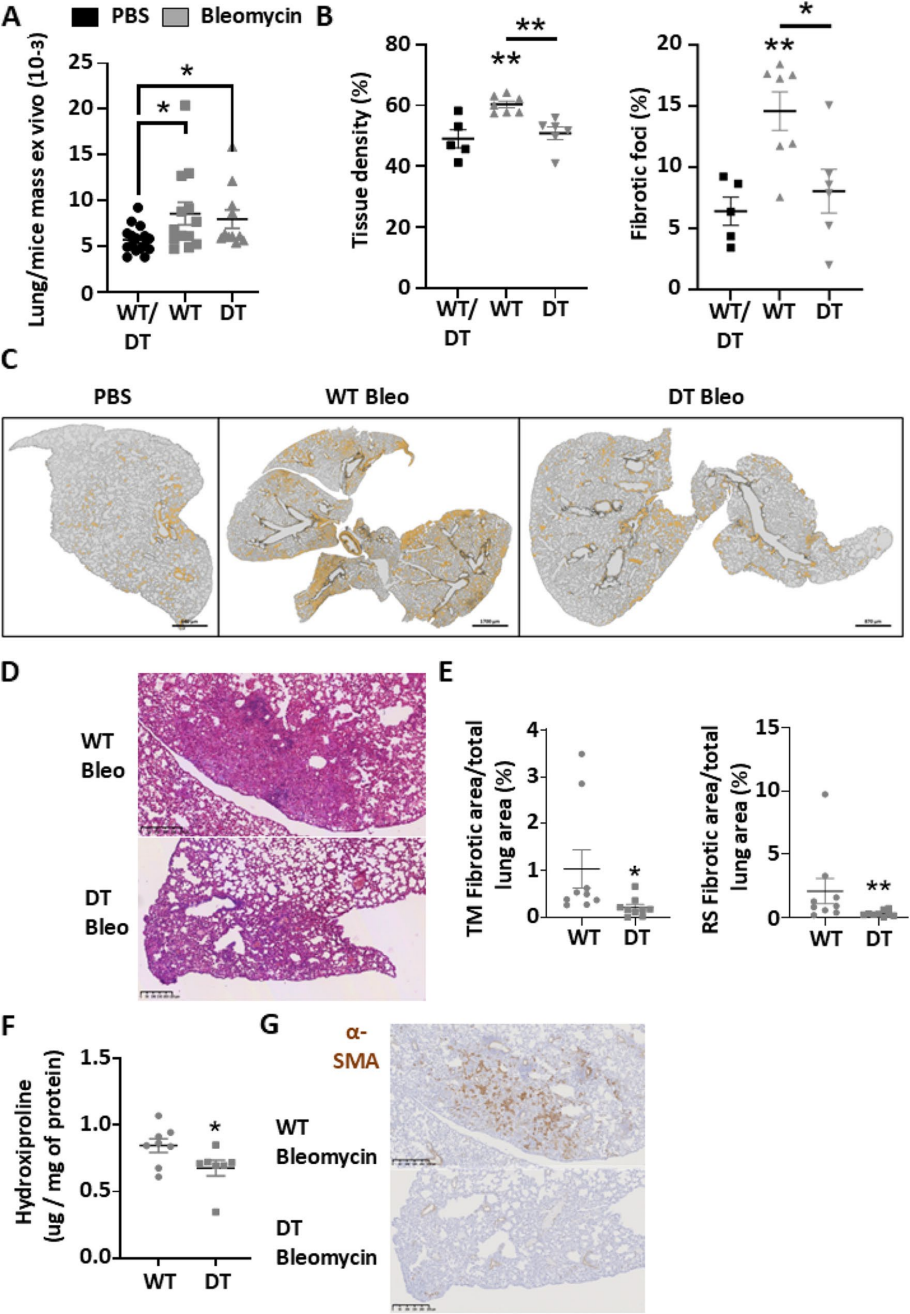
Endothelial cell human MAGI1 overexpressing mice presented attenuated lung fibrosis after systemic injections of Bleomycin. Graph summarizing lung/mice mass measurements at euthanasia (**A**). Lung sections of PBS and Bleomycin-treated wild-type (WT) and human MAGI1 overexpressing (DT) endothelial cells from mice were stained with Sirius Red. Results for lung tissue density and fibrotic foci measurements are presented in graphs (**B**). Representative images of MorphoQuant software analyses (**C**). Lung sections of PBS and Bleomycin-treated wild-type (WT) and human MAGI1 overexpressing (DT) endothelial cells from mice were stained with Masson Trichrome. Representative images and data from manual analysis of fibrotic area from Masson Trichrome and Sirius Red measurements are presented in graphs (**D**, **E**). Results from hydroxyproline assay measurements are presented in graphs (**F**). Lung sections of PBS and Bleomycin-treated wild-type (WT) and human MAGI1 overexpressing (DT) endothelial cells from mice were stained with anti-α-Smooth Muscle Actin antibody. Representative images of α-Smooth Muscle Actin-positive cells, as shown by immunostaining, are presented (**G**). Bars represent the means + SEM. The significance of differences between two groups was evaluated using the unpaired Student’s t-test or Mann–Whitney test. *P < 0.05; ** *P* < 0.01. (*n* = 13 mice PBS group/12 mice WT Bleomycin group/9 mice DT Bleomycin group)

**Fig. 4:**
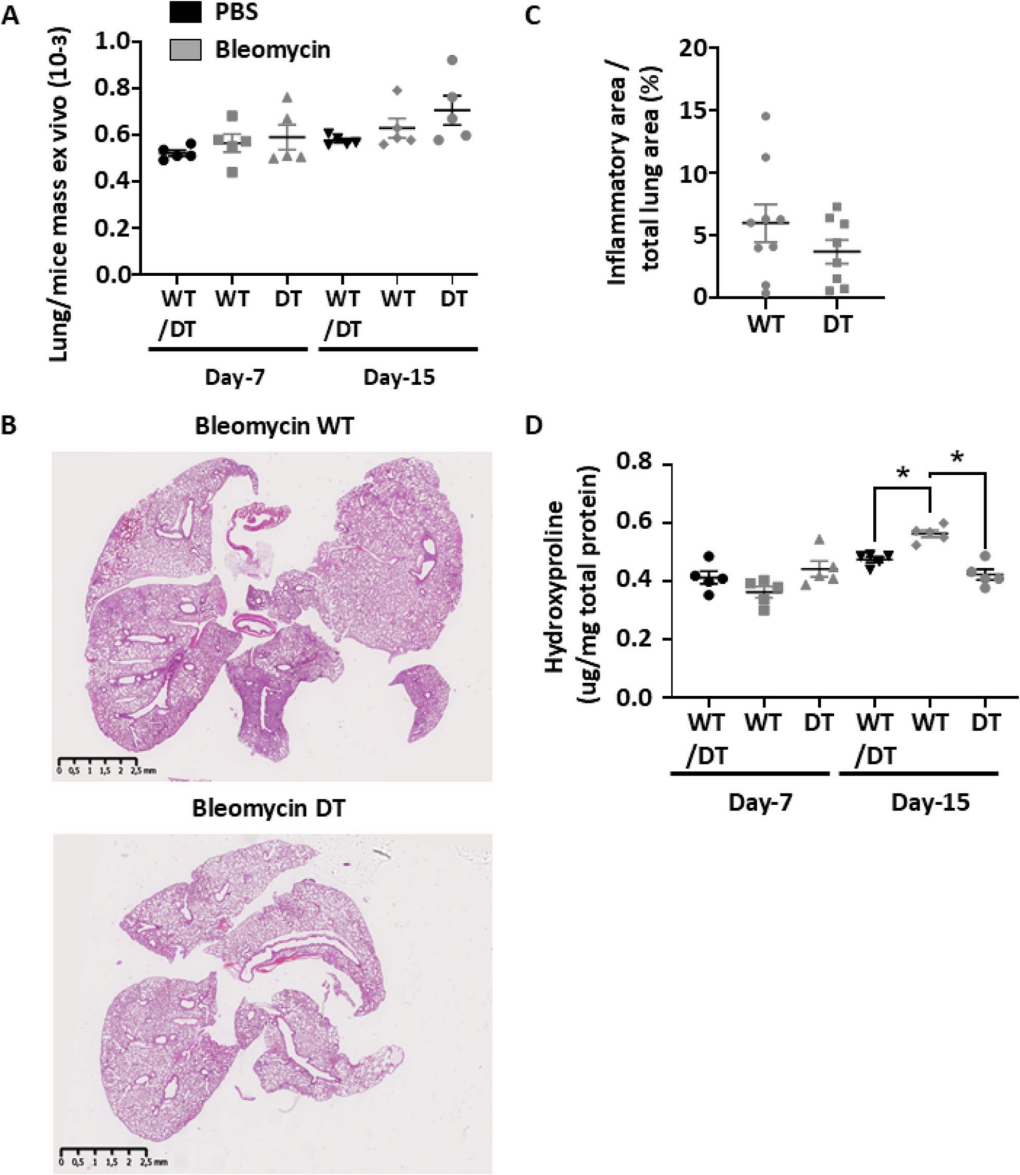
Endothelial cell human MAGI1 overexpressing mice presented attenuated tissue changes after systemic injections of Bleomycin. Graph summarizing lung/mice mass measurements at euthanasia (**A**). Lung sections of PBS and Bleomycin-treated wild-type (WT) and human MAGI1 overexpressing (DT) endothelial cells from mice were stained with hematoxylin and eosin. Results for lung tissue inflammation area measurements are presented in graphs (**B**, **C**). Results from hydroxyproline assay measurements are presented in graphs (**D**). Bars represent the means + SEM. The significance of differences between two groups was evaluated using the unpaired Student’s t-test or Mann–Whitney test. *P < 0.05. (*n* = 5 mice per group)

To evaluate the protective effect of human MAGI1 expression on endothelial cells in systemic sclerosis, we used a bleomycin-induced SSc mouse model. Experiments ended during the inflammatory phase (e.g., at days seven and fifteen) or after the development of disseminated scleroderma (e.g., day thirty-three) (Additional file 1 A). We observed that Bleomycin induced weight loss in mice during the induction period (e.g., J-1 to J-15) relative to PBS-treated mice, confirming systemic effects, while the mice regained weight after treatment was discontinued (Additional file 1B). Notably, lung weight increased over time following bleomycin treatment in both WT- and DT-treated mice, relative to PBS treatment, reaching a significant difference at day 33 (Figs. 3A and 4A). However, lung weight is similar between WT- and DT-treated Bleomycin mice.

H&E, TM, and SR coloration were performed on lung tissue sections to unravel lung density/inflammation area (e.g., H&E) and collagen fibers (e.g., TM & SR). Automated analysis revealed an increase in lung tissue density and % of fibrotic foci in Bleomycin-treated WT mice relative to PBS-treated WT mice. In contrast, Bleomycin-treated DT mice have reduced lung tissue density and a smaller area of fibrotic foci (Fig. 3B and C). Moreover, manual analysis demonstrated that Bleomycin-treated DT mice had a lower % of fibrotic tissue area than Bleomycin-treated WT mice (Fig. 3D and E). Notably, a decrease in the inflammatory area was already observed in Bleomycin-treated DT mice relative to Bleomycin-treated WT mice on day 15 (Fig. 4B and C).

Furthermore, hydroxyproline assays demonstrated a decrease in lung collagen content in Bleomycin-treated DT mice relative to Bleomycin-treated WT mice at day 33 and during the inflammatory phase at day 15 (Figs. 3F and 4D). Finally, using immunohistochemistry, we unraveled the presence of α-Smooth Muscle Actin (α-SMA) myofibroblasts in fibrotic foci of Bleomycin-treated WT mice, which is reduced in Bleomycin-treated DT mice (Fig. 3G).

These experiments confirmed that MAGI1 overexpression on endothelial cells protects from myofibroblast accumulation, fibrotic foci development, collagen deposition, and tissue densification in an animal model of systemic scleroderma.

### Human MAGI1 expression in blood vascular endothelial cells impaired macrophage recruitment in the lung fibrotic region during scleroderma development

**Fig. 5:**
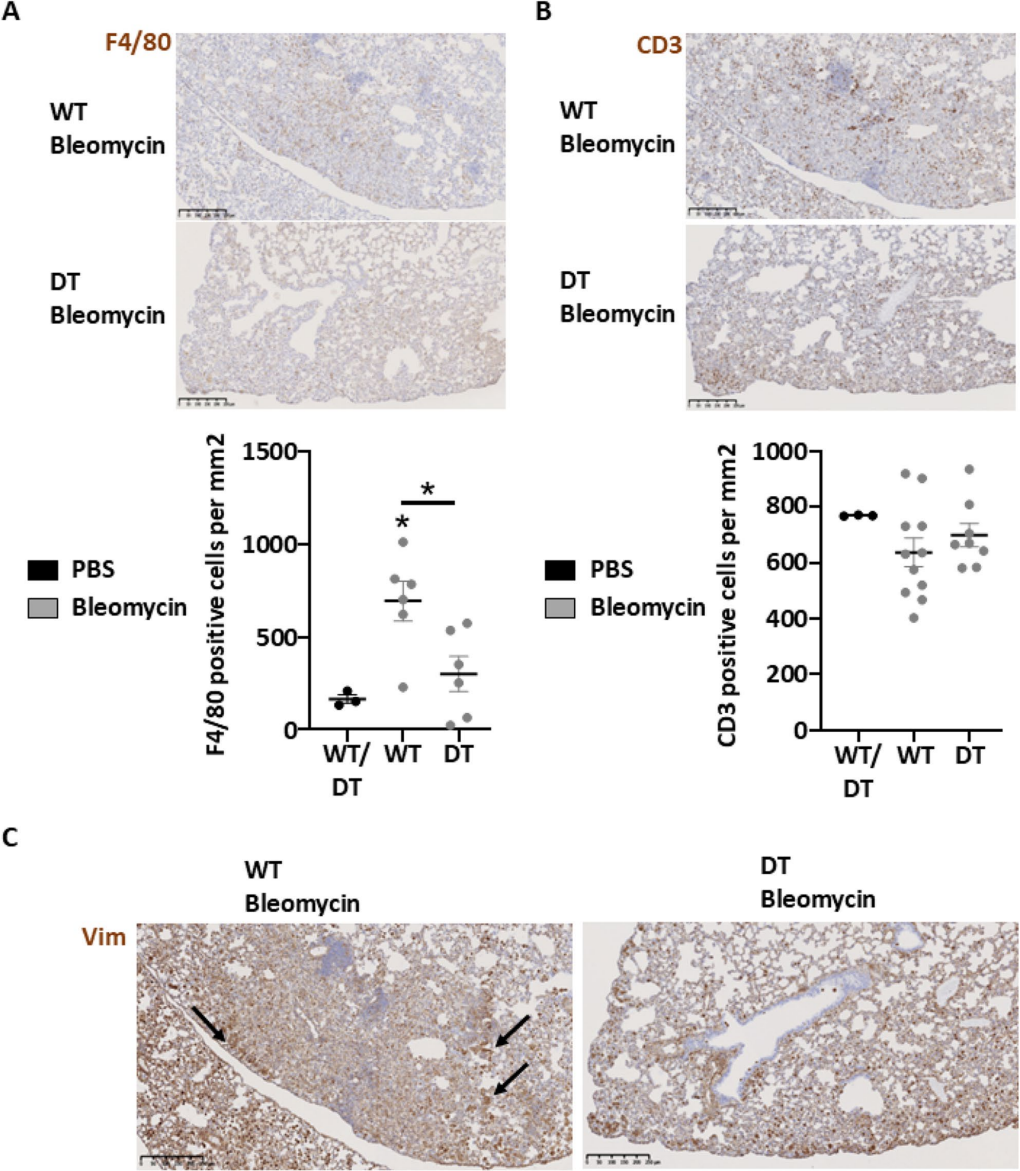
Endothelial cell human MAGI1 overexpression in mice attenuated macrophage content in systemic scleroderma lesions. Lung sections of Bleomycin-treated wild-type (WT) and human MAGI1 overexpressing (DT) endothelial cells from mice were stained with anti-F4/80, anti-CD3, and anti-Vimentin antibodies. Images of representative sections of fibrotic regions and the corresponding quantifications for F4/80^+^ (**A**), CD3^+^ (**B**), and Vimentin^+^ (**C**) cells. Bars represent the means + SEM. The significance of differences between two groups was evaluated using the unpaired Student’s t-test or Mann–Whitney test. *P < 0.05. (*n* = 3 mice PBS group/6–11 mice WT Bleomycin group/6–8 mice DT Bleomycin group)

The recruitment of pro-inflammatory and immune cells is crucial during the development of fibrosis. Accordingly, we performed immunostaining of macrophages (i.e., F4/80^+^), lymphocytes (i.e., CD3^+^), and Vimentin^+^ cells in the lung samples from the results above. We observed a decrease in F4/80^+^ macrophages in the fibrotic region of the lung of endothelial cell MAGI1 overexpressing mice relative to WT mice in SSc (Fig. 5A). However, no difference was observed in the CD3 + cells count (Fig. 5B). Moreover, the presence of Vimentin^+^ cells is also observed at the border of the lung fibrotic region WT mice. In contrast, this was not observed in endothelial cell MAGI1-overexpressing mice (Fig. 5C).

These results demonstrated that the overexpression of MAGI1 in endothelial cells reduced the recruitment of macrophages and vimentin-positive cells in the fibrotic region. At the same time, the total lymphocyte count remained unaffected during SSc development.

### Human MAGI1 expression in blood vascular endothelial cells protects from in vivo and in vitro induction of vascular permeability

**Fig. 6:**
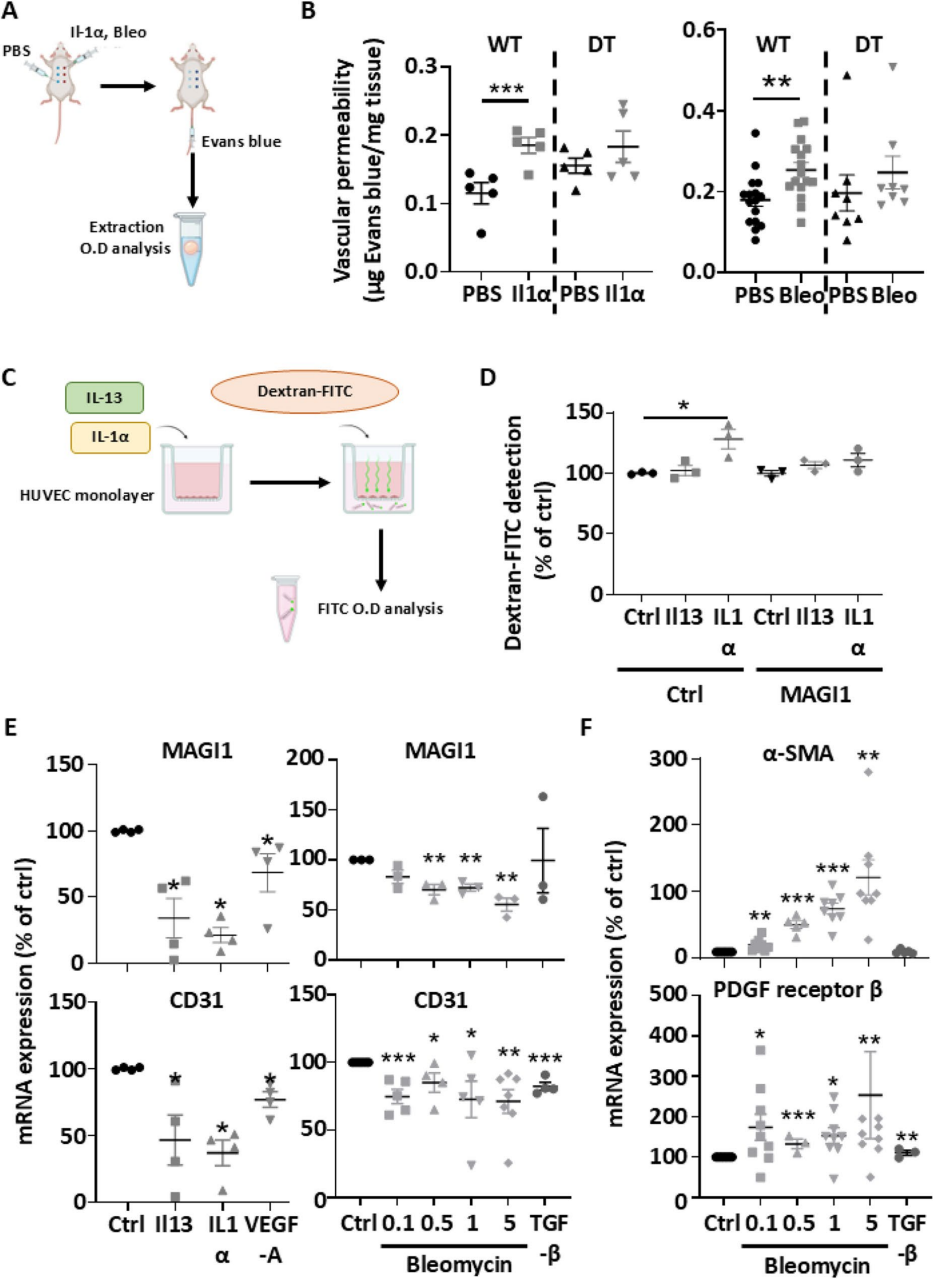
MAGI1 overexpression in HUVEC impaired the induction of vascular permeability. Scheme of experimental protocol for the Miles assay (**A**). Evans blue content in the skin was evaluated and normalized to tissue mass. Graph summarizing the measurements after IL1α and Bleomycin treatment (**B**) (Left *n* = 5 mice per group & right *n* = 16 mice WT PBS and WT Bleomycin group/*n* = 8 mice WT PBS and WT Bleomycin group). Scheme of experimental protocol for the in vitro permeability assay (**C**). Dextran FITC content was measured at the bottom of the wells after challenge with inflammatory cytokines. Graph summarizing the measurements where untreated control cells were assigned a value of 100%, and other values were set in proportion to that value (**D**) (*n* = 3). MAGI1 and CD31 mRNA levels were measured by RT-PCR and normalized to the GAPDH gene. Untreated control cells were assigned to a value of 100%, and other values were set in proportion to that value (**E**) (*n* = 4–5). PDGF receptor β and α-SMA mRNA levels were measured by RT-PCR and normalized to the GAPDH gene. Untreated control cells were assigned a value of 100%, and other values were set in proportion to that value (**F**) (*n* = 4–8). Bars represent the means + SEM. The significance of differences between two groups was evaluated using the unpaired Student’s t-test or Mann–Whitney test. *P < 0.05; ** *P* < 0.01; ***P < 0.001

The development of localized scleroderma and systemic sclerosis is highly regulated by vascular permeability [13]. In line with the results above, we hypothesize that human MAGI1 expression in endothelial cells will prevent the induction of blood vessel permeability in pro-inflammatory conditions. Accordingly, we performed an in vivo permeability assay on WT and DT mice. Blood vessel permeability was assessed by the quantification of Evans Blue extravasation in the skin of the mice after stimulation with either Bleomycin or recombinant Il-1α relative to PBS (negative control-contralateral injection) (Fig. 6A).

We found that Il-1α and bleomycin increased Evans Blue extravasation relative to PBS in WT mice, while endothelial cell human MAGI1 expressing mice (DT) do not respond to permeability inductions (Fig. 6B). Furthermore, we performed an in vitro transwell permeability assay on HUVEC monolayers to confirm these in vivo observations (Fig. 6C). After reaching full confluency, HUVEC were stimulated with pro-inflammatory cytokines (i.e., IL-1α or IL-13) for 24 h, followed by the addition of dextran-FITC dye (70 kDa) at the top of each transwell. Then, the amount of dextran-FITC in the medium at the bottom of each transwell was quantified spectrophotometrically. We observed that IL-1α induced an increased permeability in HUVEC monolayers. However, human MAGI1 overexpression in HUVEC abolished this effect (Fig. 6D).

These results confirmed that in vivo and in vitro overexpression of MAGI1 in endothelial cells protects against the induction of blood vessel permeability in response to cellular and chemical damage.

### Growth factors and pro-inflammatory cytokines downregulate MAGI1 expression in HUVECs

The activation of endothelial cells, through proangiogenic stimulation and cellular damage, has been described as an early process involved in the development of localized Scleroderma and Systemic Sclerosis. It involves multiple molecules (e.g., growth factors, interleukins, chemicals…). To determine if MAGI1 expression on endothelial cells is regulated by key molecules involved in these processes, HUVECs were stimulated with angiogenic growth factors (e.g., VEGF-A), proinflammatory cytokines (e.g., IL1α and IL13), or Bleomycin. MAGI1, endothelial-specific markers (i.e., PECAM1/CD31 and VE-Cadherin), and mesenchymal/fibroblast markers (i.e., PDGF receptor β and α-SMA) expression have been quantified at the mRNA and/or protein level. The mRNA and protein analysis revealed that HUVECs expressed MAGI1, VE-cadherin, and PECAM1/CD31 but not mesenchymal markers relative to human dermal fibroblasts (Additional file 2 A and 1B). Interestingly, we found that endothelial cell markers (i.e., MAGI1 and PECAM1) mRNA expressions are decreased after IL1α, IL13, VEGF-A, and Bleomycin treatment (Fig. 6E). Finally, we observed that mesenchymal markers (i.e., PDGF receptor β and α-SMA) mRNA expressions are increased with Bleomycin treatment (Fig. 6F).

From these experiments, we found that pro-inflammatory/damage cytokines and pro-angiogenic growth factors reduced the expression of MAGI1 and endothelial cell markers in HUVEC. Furthermore, Bleomycin treatment reduced MAGI1 and endothelial cell markers, while increasing mesenchymal marker expression in HUVECs.

### Endothelial cell MAGI1 overexpression blocks VEGF-A permeability and angiogenic properties

**Fig. 7:**
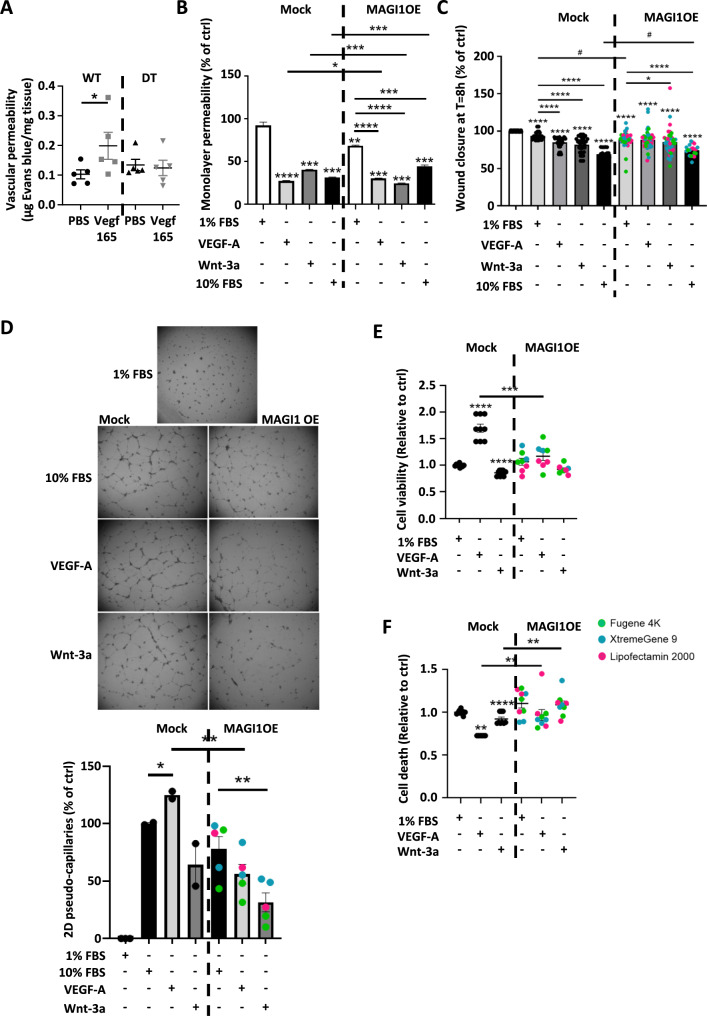
MAGI1 overexpression in HUVEC impaired the proangiogenic properties of VEGF-A. The Evans blue content in the skin was evaluated and normalized to tissue mass. Graph summarizing the measurements after VEGF-165 treatment (**A**) (*n* = 5 mice per group). Dextran FITC content was measured at the bottom of the wells after challenge with VEGF-165 and Wnt3a. Graph summarizing the measurements where untreated control cells were assigned a value of 100%, and other values were set in proportion to that value (**B**) (*n* = 3). The wound-closure property of HUVECs was measured at T = 8 h with ImageJ after challenge with VEGF-165 and Wnt3a. Graph summarizing the measurements where untreated control cells were assigned a value of 100%, and other values were set in proportion to that value (**C**) (*n* = 18). The 2D angiogenic properties of HUVECs were measured using ImageJ after challenge with VEGF-165 and Wnt3a. Representative images and a graph summarizing the measurements, where untreated control cells were assigned a value of 100%, and other values were set in proportion to that value (**D**) (*n* = 3–5). Cell viability and cell death were measured after challenge with VEGF-165 and Wnt3a. Graph summarizing the measurements where untreated control cells were assigned a value of 1, and other values were set in proportion to that value (**E**, **F**) (*n* = 8–9). Bars represent the means + SEM. The significance of differences between two groups was evaluated using the unpaired Student’s t-test or Mann–Whitney test. *P < 0.05; ** *P* < 0.01; ***P < 0.001; ****P < 0.0001

We observed that VEGF-A, a key regulator of blood vessel permeability and angiogenic function, downregulates MAGI1 expression. Moreover, it has been reported that endothelial cell MAGI1 overexpression protects against endothelial cell activation, suggesting that MAGI1 overexpression at least abolishes the activation of key signaling pathways involved in blood vessel function [17, 18]. We investigate whether MAGI1 overexpression in HUVEC influences the permeability and angiogenic properties of VEGF-A, which is known to be involved in localized and systemic scleroderma [22, 23]. We first evaluated the effects of Vegf-165 on blood vessel permeability in vivo in WT and DT mice. We observed that MAGI1 overexpression suppresses the induction of blood vessel permeability (Fig. 7A). Permeability index analysis showed that the effect is greater with Vegf-165 than with Il-1α (Additional file 3 A).

To investigate MAGI1 overexpression in vitro, HUVECs were transfected with a human MAGI1-expressing plasmid using multiple transfection reagents (i.e., Lipofectamine 2000, Fugene 4 K, and XtremeGene 9). Transfection efficiency and cellular toxicity were assessed using western blotting and the Lactate Dehydrogenase (LDH) cell death assay. A three-fold increase in MAGI1 protein expression and no toxicity were observed (Additional file 3B-3 C).

Then, we performed HUVEC stimulation with recombinant human VEGF-A and Wnt-3a in 1% FCS cell culture medium to stimulate permeability and angiogenic properties (i.e., cell migration, 2D pseudo-capillaries formation, cell viability, and cell death). We observed that MAGI1 overexpression reduced monolayer permeability, cell migration (i.e., wound healing), and pseudo-capillary formation in 10% FCS conditions (Fig. 7B and D and additional file 3D). Moreover, relative to the 1% FCS condition, VEGF-A reduced monolayer permeability, cell migration, and blocked pseudo-capillaries and cell viability/cell death protective effects of VEGF-A (Fig. 7B and E and additional file 3D-3E). Furthermore, MAGI1 overexpression also suppressed the induction of Wnt3a pseudo-capillaries and the protective effects against cell death (Fig. 7D and E).

These results demonstrated that MAGI1 overexpression in endothelial cells suppressed VEGF-A pro-angiogenic (i.e., cell viability, cell death, cell migration, and pseudo-capillary properties) and induced permeability effects. Moreover, it suppressed Wnt3A-dependent induction of pseudo-capillaries.

## Discussion

In this study, we were able to demonstrate that: 1/ MAGI1 overexpression on endothelial cells protects from localized and systemic sclerosis in the lung using three animal mice models, 2/ the protective effect involves a decrease in skin thickness, tissue damage, collagen deposition, fibrotic foci in the skin and lung, 3/ at a cellular level, a decreased in α-SMA^+^ myofibroblasts and F4/80^+^ macrophages is observed in the tissue, 4/ MAGI1 expression on endothelial cells is reduced by angiogenic and inflammatory/chemical stimulus in vitro, and 5/ MAGI1 overexpression in endothelial cells protects from VEGF-A pro-angiogenic and permeability induction.

Despite extensive investigations into cancer and epithelial cells, especially in the context of tumors, the role of MAGI1 in angiogenic pathology and related events remains relatively underexplored [14–24]. In *both in vivo* and pathological conditions, a strong association has been observed between the MAGI1 locus and chronic inflammatory diseases, such as Crohn’s disease and psoriasis. At the same time, MAGI1 regulates osteoclast fusion, which is a key process in inflammation and degeneration associated with osteoarthritis. Finally, MAGI1 is involved in the inflammatory response to influenza A virus (IAV), potentially promoting the infection and the development of cardiovascular disease by activating endothelial cells. We used an in vivo mouse transgenic model to investigate human MAGI1 expression in endothelial cells [17, 18] under pathologic angiogenic conditions, with a focus on LSc and SSc.

The extent of LSc and SSc in animal models can vary depending on the strain, sex, age, and chemicals used. Accordingly, we performed experiments using multiple LSc and SSc models in WT and human MAGI1-overexpressing endothelial cell mice. We observed that MAGI1 overexpression protects against LSc induced by intradermal injection of Bleomycin and hypochlorite acid, as well as against SSc induced by systemic injection of Bleomycin. Interestingly, the sodium hypochlorite model demonstrates a greater ability to induce LSc than Bleomycin, at least at the dose and time frame tested. The Bleomycin LSc model appears to represent an intermediate state, with slight modifications in tissue morphology but no increase in collagen deposition. Moreover, within the time frame and doses used for this study, lung fibrosis was rarely observed and therefore was not analyzed in these two models; instead, it was investigated in the systemic scleroderma models. Using the systemic scleroderma model, we observed lung fibrosis almost exclusively in male mice. Better results were achieved when mice were at least 10–12 weeks old, with a complete hormonal system, as observed in other genetic backgrounds [25]. The FVB/N background used in this model is partially resistant to intradermal or systemic doses of Bleomycin, yet we still obtained significant results. Due to the 3Rs constraint, we did not perform complementary experiments by increasing the dose or the number of injections in mice, as this would have increased stress and reduced the animal’s quality of life.

Using these models, we found that LSc is reduced in endothelial cell MAGI1-overexpressing mice, which is associated with a decrease in skin layer thickness and collagen deposition in the tissue. Moreover, SSc development was also reduced in the lungs. SSc is associated with reduced tissue density, the development of fibrotic foci, inflammation, and collagen deposition. Notably, these results were obtained through classical manual analysis and biochemical assays and subsequently confirmed using Automated Analysis Software validated for the detection of lung fibrosis [26]. In association with changes in tissue architecture, MAGI1 overexpression in endothelial cells reduced the content of macrophages and myofibroblasts in lung fibrotic foci. No differences were observed in CD3 T lymphocyte staining, indicating that a deeper characterization of lymphocyte subtypes or other inflammatory and immune cells (e.g., neutrophils, basophils, mast cells) can be extended in the future. Indeed, CD3^+^ T lymphocytes account for the total lymphocyte count, whereas analysis of either the B or T lymphocyte subpopulations, including Th1, Th2, and Th17, would provide additional value and a more precise assessment of the immune response regulated by MAGI1. These experiments will be conducted in the future, using immunostaining alongside RNA sequencing on tissue samples.

The recruitment of inflammatory cells and the development of fibrosis are regulated by blood vascular endothelial cells, and the pro-angiogenic factor VEGF-A plays a crucial role in these processes. The role of endothelial cells in the development of fibrosis can be direct, involving the endothelial to mesenchymal transition (endoMT), constituting a direct source of myofibroblast, or indirectly through the secretion of pro-fibrotic and inflammatory mediators, blood vessel leakiness, cellular senescence/Secretory Associated Senescence Phenotype, and impairment of tissue repair mechanisms [27–30]. Interestingly, VEGF-A, as well as tissue damage-associated cytokines (i.e., IL-1α and IL-13) and chemicals (i.e., bleomycin), reduced MAGI1 expression in endothelial cells. These results highlight the critical role of MAGI1 in healthy endothelial cells, suggesting that its regulation may contribute to the progression of vascular and inflammatory diseases. Accordingly, we found that MAGI1 overexpression in endothelial cells, both in vitro and in vivo, protects against VEGF-A- and cytokine/chemical-induced long-term changes in permeability, which are known to be associated with tissue changes, inflammation, and fibrosis [31–33]. Moreover, endothelial cell angiogenic properties (i.e., proliferation, migration, and invasion) are overactivated and dysregulated in pathological conditions, in part due to activation of the VEGF-A signaling pathway [34]. Consequently, we found that MAGI1 overexpression in HUVECs suppressed the angiogenic properties of VEGF-A, in line with previous results [18]. In addition, it also blocked the pro-angiogenic properties of Wnt3A. The Wnt/β-Catenin pathway is known to regulate VEGF function and endothelial cell angiogenic properties, and β-Catenin is a well-known partner of MAGI1. These last results highlight the potential for MAGI1 overexpression to regulate multiple pathways involved in endothelial cell dysfunction. It is important to note that, because of their origin (e.g., embryonic cells), the effects of MAGI1 overexpression and the cellular responses to growth factors and experimental conditions may differ in primary endothelial cells. For this reason, it would be valuable in the future to conduct experiments on primary endothelial cells from a specific tissue of interest.

Interestingly, MAGI1 expression is reduced by pro-inflammatory (IL-13 and IL-1α) and pro-angiogenic/permeability stimuli such as VEGF-A. This can be linked to research demonstrating that IL-13 is associated with the inflammation and fibrosis characteristic of localized and systemic scleroderma. Moreover, in patients with localized scleroderma, elevated serum levels of IL-13 have been observed compared to healthy controls [35, 36]. Furthermore, the pro-inflammatory cytokine IL-1α is associated with localized scleroderma (morphea) and systemic sclerosis (SSc) due to its role in driving fibrosis. Interestingly, unlike IL-13, scleroderma serum levels in localized scleroderma might not differ from those of healthy individuals. However, treated patients show changes in IL-1α and other IL-1 family cytokines. Finally, IL-1α promotes the differentiation of fibroblasts into myofibroblasts, increases collagen production, and can lead to skin thickening —a hallmark of both localized and systemic scleroderma [37–39].

These results highlight a new role for MAGI1, despite its role in tumor development (e.g., regulation of Epithelial-Mesenchymal Transition (EMT) and cancer cell motility/metastatic ability and inhibition of Wnt/β-catenin signaling) [14, 24].

In addition to these results, it would be interesting to investigate in the future at which level MAGI1 blocks the VEGF-A signaling pathway. It could be either directly through VEGF receptor activity/expression or indirectly through mediators and partners of the VEGF receptors, such as VE-Cadherin. Indeed, VE-cadherin is a partner of both MAGI1 and VEGFR2, and its expression is decreased in systemic sclerosis (SSc). Increased VE-cadherin expression is not currently a barrier to systemic sclerosis (SSc); instead, loss of VE-cadherin is a characteristic feature of the disease that contributes to vascular damage and dysfunction [40, 41]. Studies suggest that restoring VE-cadherin levels, which occur during successful therapy, is associated with improved outcomes, such as capillary regeneration [42]. Therefore, blocking SSc would involve restoring VE-cadherin to normal levels rather than increasing it, thereby helping stabilize the endothelium and improve vascular function. The same investigation could be performed with MAGI1 on patient samples. A limitation of this study is the lack of patient sample data, which will be collected in the future.

This study demonstrates that endothelial cell MAGI1 overexpression in mice and in vitro in endothelial cells displayed protective effects against pathologic angiogenesis and the induction of blood vessel permeability induced by VEGF-A. Furthermore, it also protects from the development of tissue damage and fibrosis recruitment in chronic conditions such as Localized and Systemic Scleroderma.

## Supplementary Information

Supplementary Material 1.

Supplementary Material 2.

## Data Availability

The datasets used and/or analyzed during the current study are available from the corresponding author on reasonable request.
