# Supplementary material for: Human MAGI1 expression in endothelial cells protects from the development of localized and systemic scleroderma in mice
Source: Arthritis Res Ther. 2026 Mar 11;28:92. doi: 10.1186/s13075-026-03777-y (PMC13094054; doi:10.1186/s13075-026-03777-y)
Supplement: Supplementary file 1 — Supplementary Material 1. [file 13075_2026_3777_MOESM1_ESM.pdf]

Additional Fig. 1. Timeline and monitoring of mice weight after induction of systemic scleroderma using Bleomycin. Scheme of experimental timeline for the systemic scleroderma models (A). Graph summarizing % of mice mass loss at euthanasia (n=5-9 mice per group)(B). Bars represent the means + SEM. The significance of differences between two groups was evaluated using the unpaired Student's t-test or Mann–Whitney test. *P < 0.05; ** P < 0.01; ***P < 0.001.

Additional Fig. 2. Comparison of endothelial and mesenchymal markers expression in HUVECs and dermal fibroblasts. MAGI1, CD31, VE-Cadherin, and PDGF receptor β mRNA expression was measured in HUVECs and dermal fibroblasts using RT-PCR and normalized to the GAPDH gene (A) (n=4). MAGI1, α-SMA, and PDGF receptor β protein expression were detected in HUVECs and dermal fibroblasts by western blot experiments. GAPDH and β-actin were used as internal controls (B)(n=3). Bars represent the means + SEM.

Additional Fig. 3. MAGI1 overexpression in endothelial cells blocks permeability induction in vivo and angiogenic properties in vitro. Permeability indexes were calculated and represented as percentages from the Miles assay in WT and DT mice challenged with IL-1α and VEGF165 (A)(n=5 mice per group). MAGI1 expression was detected in HUVECs after transfection with a plasmid encoding human MAGI1. GAPDH and β-actin were used as internal controls. Graph summarizing MAGI1 protein expression. (B)(n=5). Cell death was measured after transfection of the plasmid encoding human MAGI1. Graph summarizing the measurements where mock-transfected control cells were assigned a value of 1, and MAGI1 values were set in proportion to that value (C)(n=8-9). Representative brightfield microscopic images of wound healing assays of Mock and human MAGI1 encoding plasmid-transfected HUVECs (D). Representative brightfield microscopic images of HUVECs after transfection of plasmid encoding Mock or human MAGI1 (E). Bars represent the means + SEM. The significance of differences between two groups was evaluated using the unpaired Student's t-test or Mann–Whitney test. *P < 0.05; ** P < 0.01; ***P < 0.001.
