# Supplementary figures and images for: Human MAGI1 expression in endothelial cells protects from the development of localized and systemic scleroderma in mice
Source: Arthritis Res Ther. 2026 Mar 11;28:92. doi: 10.1186/s13075-026-03777-y (PMC13094054; doi:10.1186/s13075-026-03777-y)

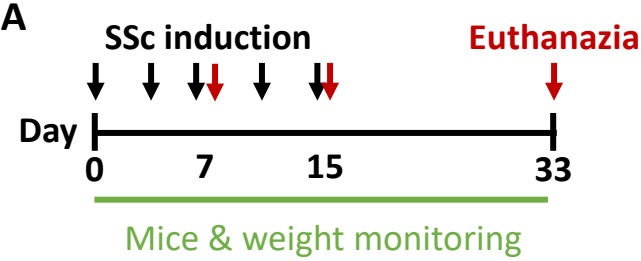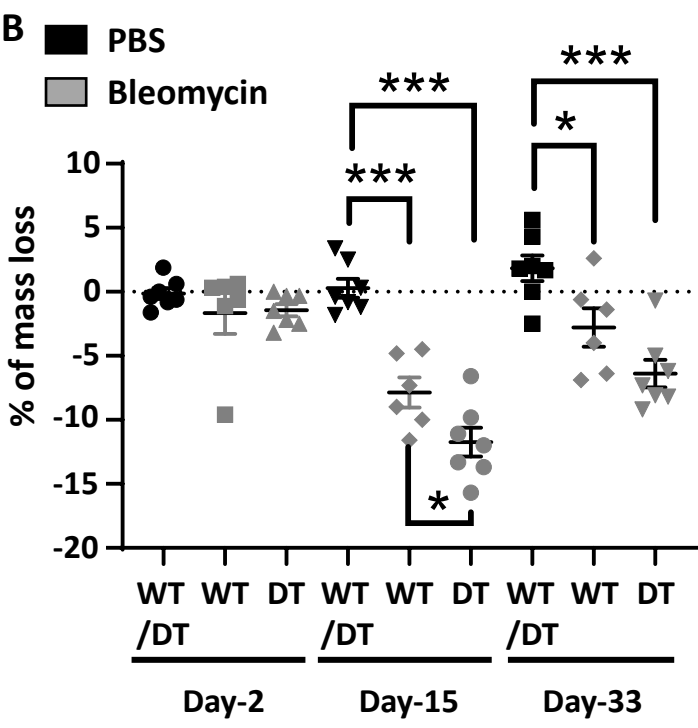

A

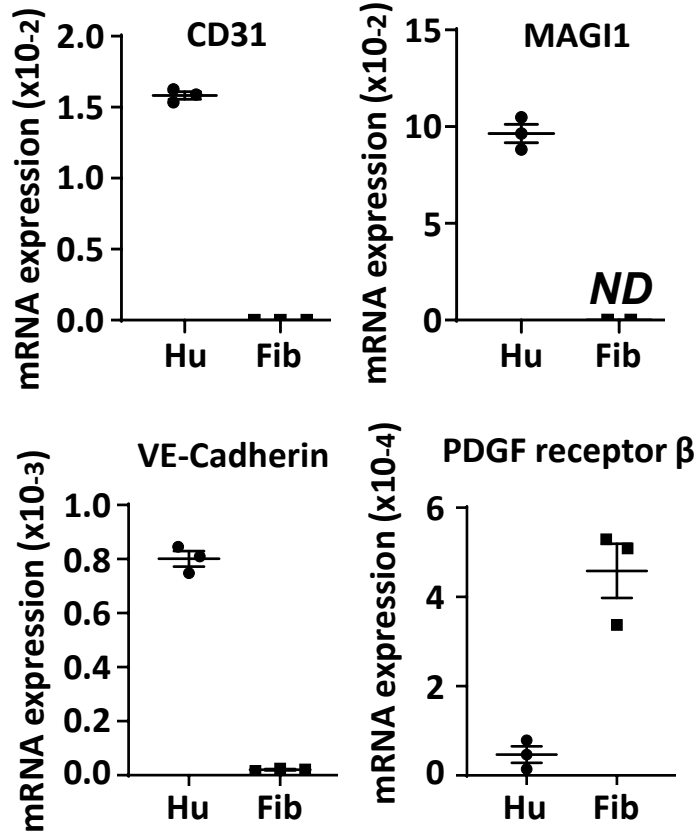

B

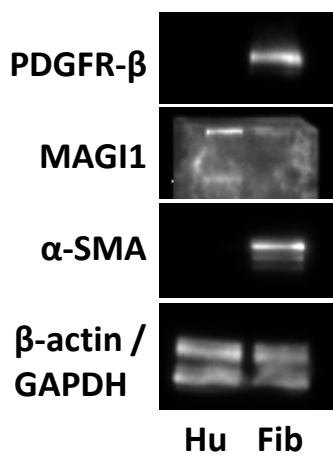

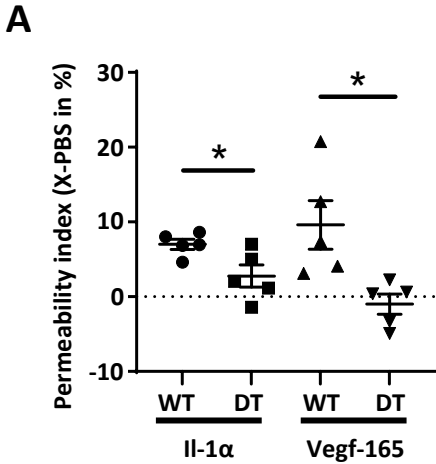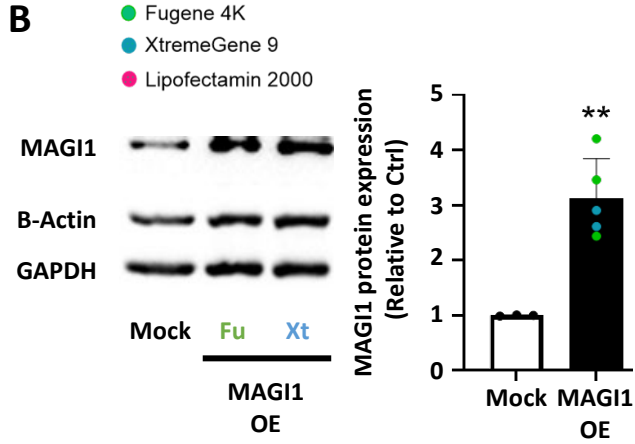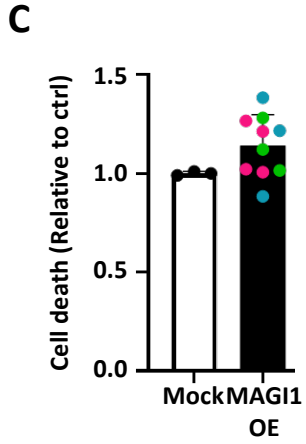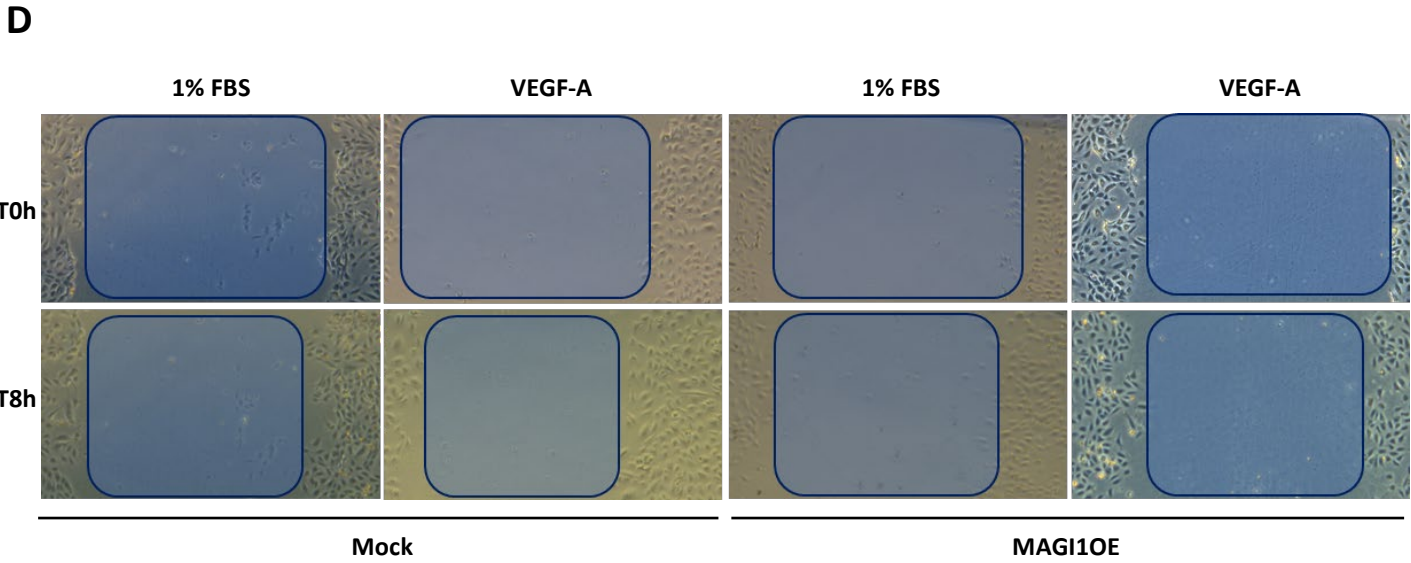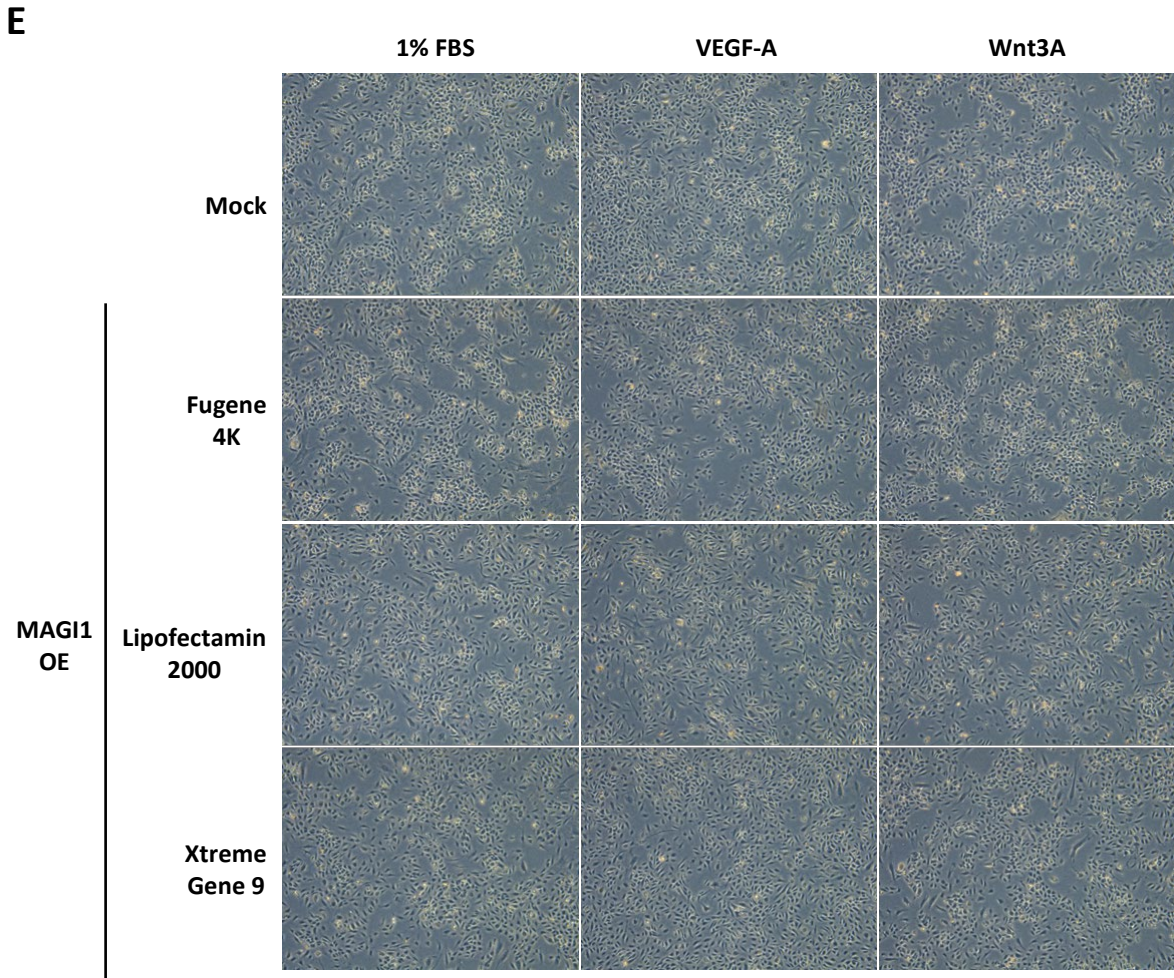

Supplement: Supplementary file 2 — Supplementary Material 2. [file 13075_2026_3777_MOESM2_ESM.pdf]
